# Effect of febuxostat on the level of malondialdehyde‐modified low‐density lipoprotein, an oxidative stress marker: A subanalysis of the PRIZE study

**DOI:** 10.1002/clc.24014

**Published:** 2023-03-29

**Authors:** Hiroki Teragawa, Atsushi Tanaka, Yuichi Fujii, Hisako Yoshida, Tomohiro Ueda, Shuichi Nomura, Toshiaki Kadokami, Hisashi Koide, Makoto Saito, Hiroaki Sano, Yasuko K. Bando, Toyoaki Murohara, Koichi Node, for the PRIZE Study Investigators

**Affiliations:** ^1^ Department of Cardiovascular Medicine JR Hiroshima Hospital Hiroshima Japan; ^2^ Department of Cardiovascular Medicine Saga University Saga Japan; ^3^ Department of Medical Statistics Osaka Metropolitan University Graduate School of Medicine Osaka Japan; ^4^ Department of Cardiovascular Medicine Saiseikai Futsukaichi Hospital Fukuoka Japan; ^5^ Department of Diabetes, Metabolism and Endocrinology Chiba University Hospital Chiba Japan; ^6^ Department of Internal Medicine Nishio Municipal Hospital Nishio Aichi Japan; ^7^ Department of Cardiology Nagoya Ekisaikai Hospital Nogaya Aichi Japan; ^8^ Department of Cardiology Nagoya University Graduate School of Medicine Nagoya Aichi Japan

**Keywords:** antioxidative effect, febuxostat, malondialdehyde‐modified low‐density lipoprotein, oxidative stress marker

## Abstract

**Background:**

Febuxostat is a selective xanthine oxidase inhibitor that reportedly exhibits antioxidant properties. We previously performed a multicentre, randomized controlled (PRIZE) study for vascular evaluation under uric acid (UA) control by febuxostat to investigate the progression of carotid lesions in asymptomatic hyperuricemic patients with carotid atherosclerosis for 2 years.

**Hypothesis:**

The current subanalysis of the PRIZE study aimed to assess the effect of febuxostat on the level of malondialdehyde‐modified low‐density lipoprotein (MDA‐LDL), an oxidative stress marker.

**Methods:**

We recruited 383 patients (febuxostat group, *n* = 200; control group, *n* = 183) from the PRIZE trial for whom MDA‐LDL measurements were available. The UA, MDA‐LDL, low‐density lipoprotein cholesterol (LDL‐C) levels, and MDA‐LDL/LDL‐C ratio were identified, represented as the estimated difference from baseline to 24 months. We also evaluated the relationship between febuxostat dose (10, ≤20 to <40, and ≤40 to ≤60 mg) and changes in the MDA‐LDL level, LDL‐C level, or MDA‐LDL/LDL‐C ratios.

**Results:**

The estimated change in MDA‐LDL/LDL‐C ratio from baseline to 24 months was significantly lower in the febuxostat group than in the control group (*p* = .025), whereas the estimated changes in MDA‐LDL (*p* = .235) and LDL‐C (*p* = .323) levels did not differ between the two groups. No significant correlation existed between the febuxostat doses and the estimated change in the MDA‐LDL level (*p* = .626), LDL‐C level (*p* = .896), or MDA‐LDL/LDL‐C ratio (*p* = .747).

**Conclusions:**

Our findings may indicate a possibility that febuxostat can lower the MDA‐LDL/LDL‐C ratio, a potential marker of atherosclerosis and oxidative stress, in asymptomatic hyperuricemic patients with carotid atherosclerosis. Further studies are required to validate our findings and elucidate the clinical antioxidant effect of febuxostat.

## Graphical abstract

IMT, intima‐media thickness; LDL‐C, low‐density lipoprotein cholesterol; MDA‐LDL, malondialdehyde‐modified low‐density lipoprotein; UA, uric acid.

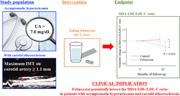

## 1 INTRODUCTION

Oxidative stress may expedite the development of atherosclerosis, which is mediated, in part, by endothelial dysfunction, by triggering the inactivation of nitric oxide^1^, ^2^, ^3^, ^4^ and/or oxidation of proteins and lipid peroxidation of membrane polyunsaturated fatty acids in lipoproteins by reactive oxygen species (ROS). Accordingly, the application of pharmacological antioxidants, nutritional antioxidants like vitamin C or E, and beta‐carotene to inhibit atherosclerosis progression has garnered interest.^5^

Oxidative stress can be analyzed in three ways: (1) by directly measuring ROS, (2) by measuring the presence or absence of antioxidants, or (3) by measuring damage to proteins, lipids, DNA, or RNA. Nevertheless, because ROS is highly unstable, the damage to proteins, lipids, DNA, or RNA, is often quantified to assess oxidative stress in clinical practice. Malondialdehyde (MDA)‐modified low‐density lipoprotein (LDL) is LDL modified by MDA, which is a large and structurally distinct aldehyde formed when LDL undergoes oxidative denaturation; it is one of the representative markers of oxidized LDL, which is a general term for various substances produced by oxidative denaturation of LDL. This oxidative stress marker is often used in clinical practice.^6^, ^7^, ^8^

Febuxostat selectively suppresses xanthine oxidase (XO), the enzyme responsible for the conversion of hypoxanthine to xanthine and then to uric acid (UA), thereby reducing the UA levels. Hence, this drug has an antioxidant effect,^6^, ^9^, ^10^, ^11^, ^12^, ^13^, ^14^, ^15^, ^16^, ^17^, ^18^, ^19^, ^20^ which was shown to be robust in various basic and animal studies.^9^, ^10^, ^11^, ^13^, ^15^, ^17^, ^18^, ^19^ Many clinical studies have also demonstrated the antioxidant effects of febuxostat^6^, ^12^, ^14^, ^16^, ^20^, ^21^; however, the results are inconsistent. Sezai et al. stated that, in hyperuricemic patients with cardiovascular disease treated with a combination of febuxostat and topiroxostat, the oxidized LDL level was significantly lower in the febuxostat group at 3 months, but the difference disappeared at 6 months.^16^ Contrarily, Sezai et al. also reported that febuxostat significantly reduced the oxidized LDL levels at 6 months compared with allopurinol in hyperuricemic patients undergoing cardiac surgery, although the difference was not significant at 3 months.^12^ Tausche et al. indicated that, in patients with gout, febuxostat did not alter the levels of thiobarbituric acid‐reactive substances or MDA‐LDL at 12 months, but it significantly reduced the nicotinamide adenine dinucleotide phosphate oxidase activity at 12 months.^14^ Suzuki et al. tated that 8‐hydroxy‐2′‐deoxyguanosine, an index of oxidative stress, was significantly decreased in patients with heart failure, particularly in those with preserved left ventricular ejection fraction, after 3 years of febuxostat treatment.^20^ Conversely, to date, few studies have compared antioxidant effects in hyperuricemic patients with atherosclerotic lesions, as frequently experienced in daily practice, with a control group over a 2‐year observation period.

In a recent multicentre, randomized controlled (PRIZE) study, vascular evaluation was conducted under UA control by febuxostat.^22^ The investigation demonstrated that febuxostat did not affect the progression of carotid atherosclerosis, evaluated as intima–media thickness (IMT) for 2 years. In the current post hoc subanalysis of the PRIZE study, to validate the antioxidative effect of febuxostat in a clinical setting, we examined the influence of febuxostat on the serum MDA‐LDL level. We also assessed whether febuxostat and MDL‐LDL levels have a dose‐dependent relationship.

## 2 METHODS

### 2.1 Trial design and participants

The PRIZE study was a multicentre, prospective, randomized, open‐label, blinded‐endpoint clinical experiment. The specifics of the study rationale and design have been previously reported.^23^ After trial registration was completed in January 2014 (University Hospital Medical Information Network Clinical Trial Registry, UMIN000012911), trial recruitment was carried out between May 2014 and August 2018 at 48 clinical sites throughout Japan. After their eligibility was validated and their medical backgrounds reviewed, patients were randomized equally to either the febuxostat (patients receiving 10–60 mg febuxostat daily) or (patients receiving nonpharmacological treatment of hyperuricaemia) control groups. All patients were followed up with study visits scheduled at 1, 2, 3, 6, 12, and 24 months after the baseline visit. Carotid artery ultrasonography for the main evaluation and blood sampling was conducted at baseline and after 12 and 24 months (or at premature termination) at each local site.

The trial was performed in full compliance with the Declaration of Helsinki and following the Ethical Guidelines for Medical and Health Research Involving Human Subjects established by the Ministry of Health, Labour and Welfare and the Ministry of Education, Culture, Sports, Science, and Technology in Japan. Before enrollment, all participants received a sufficient explanation of the research plan and gave written informed consent.

The current subanalysis was secondarily planned and conducted after the publication of the main investigation in the PRIZE study,^22^ newly registered as UMIN000041322.

### 2.2 Randomization and intervention

The inclusion and exclusion criteria of the research were previously stated.^22^, ^23^ Briefly, adults (aged ≥20 years) with asymptomatic hyperuricaemia were eligible. Enrolled patients had a serum uric acid (SUA) of >7.0 mg/dL and a maximum common carotid artery (CCA) IMT of ≥1.1 mm, measured at eligibility assessment, described as a carotid arterial plaque (localized protruding lesion). The main exclusion criteria were the administration of SUA‐lowering agents within the 8 weeks before eligibility analysis, the presence of gouty tophus, or symptoms of gout arthritis occurring within 1 year before the eligibility test.

**Figure 1:**
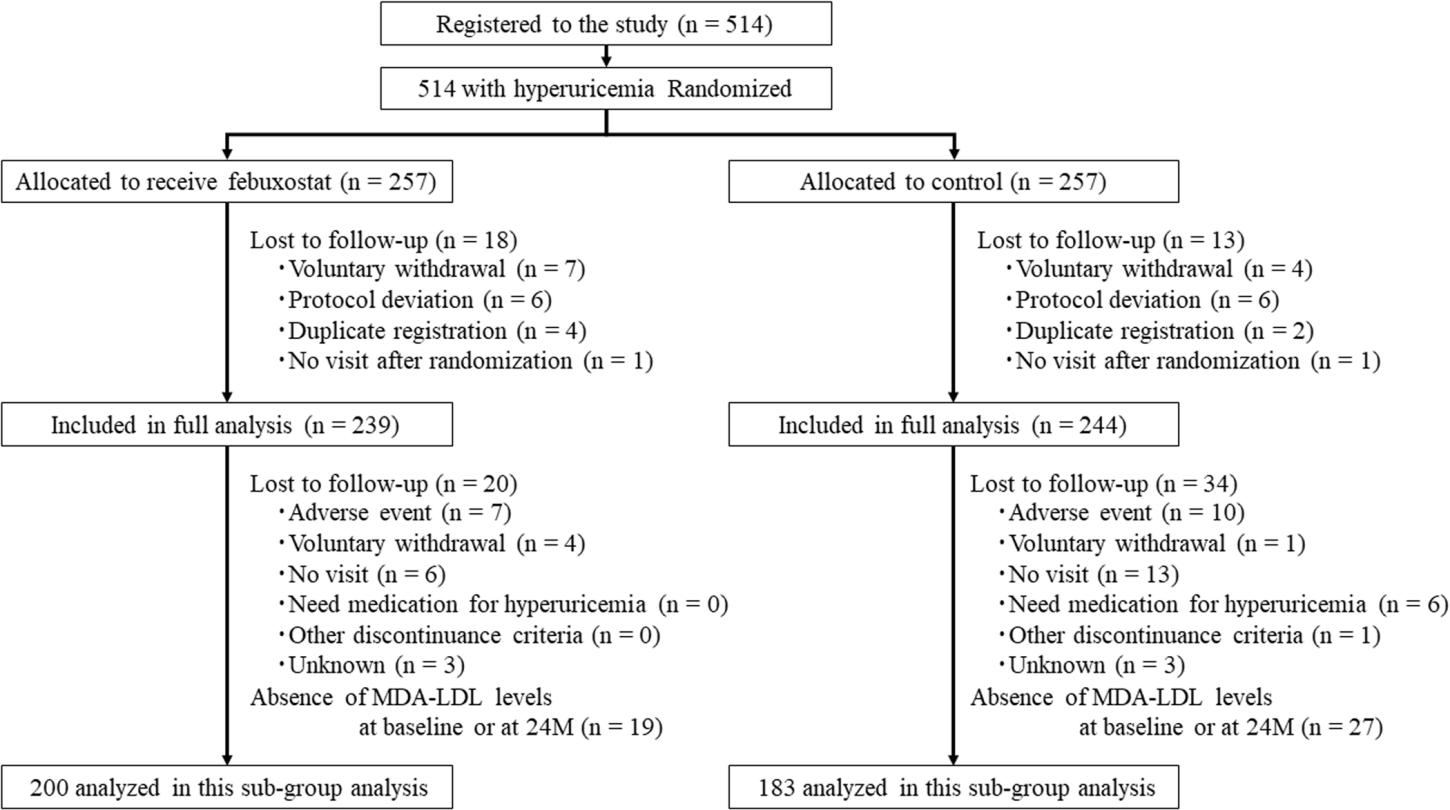
Study flowchart. M, months; MDA‐LDL, malondialdehyde‐modified low‐density lipoprotein.

The current subanalysis involved 383 patients with available data about MDA‐LDL levels both at baseline and at the end of observation at 24 months (febuxostat group, *n* = 200; control group, *n* = 183; Figure 1). Patients were randomized to the febuxostat or control groups in a 1:1 ratio at the automatic web‐based PRIZE Data Centre, as stated previously.^23^ All participants in both groups were prescribed suitable lifestyle modifications for hyperuricemia, such as a healthy diet and exercise therapy, which was as follows: exercise therapy at least three times a week for 30 min/day and energy optimization to eradicate obesity and overweight, dietary guidance such as limiting alcohol consumption, improving excessive intake of carbohydrates, meat, and seafood, and recommending consumption of DASH diet, Mediterranean diet, and fruit and soy diet. These instructions were given using a pamphlet, and modifications were regulated during the study period. Following the protocol, the febuxostat group received an initial dose of 10 mg daily that was titrated to 20 mg daily in the first month and 40 mg daily in the second month, with a target maintenance dose of 40 mg. Nevertheless, at 3 months or later, the dose of febuxostat could be increased to 60 mg daily. If the SUA levels declined to ≥2.0 mg/dL during the study period, the maintenance dose was decreased by 20 mg.

Each participant's baseline treatment, including antidiabetic, antiplatelet, antihypertensive, and lipid‐lowering agents, remained unaffected during the study period, if possible, based on the patient's clinical condition.

### 2.3 Measurements

Blood samples were obtained at baseline and after 24 months, with the exception of the UA level, which was measured at baseline, 6, 12, and 24 months. The SUA and LDL‐cholesterol (LDL‐C) concentrations were quantified at each local site, and the MDA‐LDL level was measured at a centralized laboratory (SRL Co.). For the measurement of MDA‐LDL levels, an enzyme‐linked immunosorbent assay (Oxidized LDL ELISA Daiichi; SEKISUI) was employed.^24^ The levels of SUA (mg/dL), MDA‐LDL (U/L), and LDL‐C (mg/dL) were assessed in the present subanalysis. Alterations in UA level from baseline to 6, 12, and 24 months and those in the MDA‐LDL level, LDL‐C level, and MDA‐LDL/LDL‐C ratio from baseline to 24 months were quantified in the two groups. The estimated changes in MDA‐LDL level, LDL‐C level, and MDA‐LDL/LDL‐C ratio at 24 months from baseline were also measured in the two groups. Additionally, the association between the final dose of febuxostat taken and the percentage changes in MDA‐LDL level, LDL‐C level, and MDA‐LDL/LDL‐C ratio were analyzed in the present subanalysis.

The detailed protocol and procedure for measuring carotid IMT were described previously.^22^, ^23^, ^25^ The mean far‐wall CCA‐IMT on the left and right sides was averaged, and the estimated change in mean CCA‐IMT values at 24 months from baseline was measured. We evaluated the association between the estimated change in mean CCA‐IMT and those in MDA‐LDL level, LDL‐C level, and MDA‐LDL/LDL‐C ratio.

### 2.4 Statistical analysis

All efficacy analyses were performed in a modified intention‐to‐treat manner, comprising all randomized participants not lost to observation. For the baseline variables, the summary statistics were denoted as frequencies and proportions for categorical data and median (interquartile range) for continuous variables. The estimated change in the MDA‐LDL level, LDL‐C level, and MDA‐LDL/LDL‐C ratio from baseline to 24 months (including 95% confidence intervals) were analyzed using an analysis of covariance. This model assessed the difference in the trends from baseline to each time point; the treatment groups were modified for age, sex, UA concentration, and baseline MDA‐LDL level, LDL‐C level, and MDA‐LDL/LDL‐C ratio. The outcome variable was natural‐logarithm transformed to satisfy the assumption of error term normality. To illustrate the relationships between changes in UA level, MDA‐LDL level, LDL‐C level, or MDA‐LDL/LDL‐C ratio, linear regression analyses, modified for each value at baseline, were conducted. All *p* values were two‐sided with a level of significance of .05, and there were no adjustments for multiple comparisons. All statistical analyses were carried out utilizing R version 4.0.3 (https://cran.r-project.org/).

## 3 RESULTS

### 3.1 Patient characteristics

**Figure 2:**
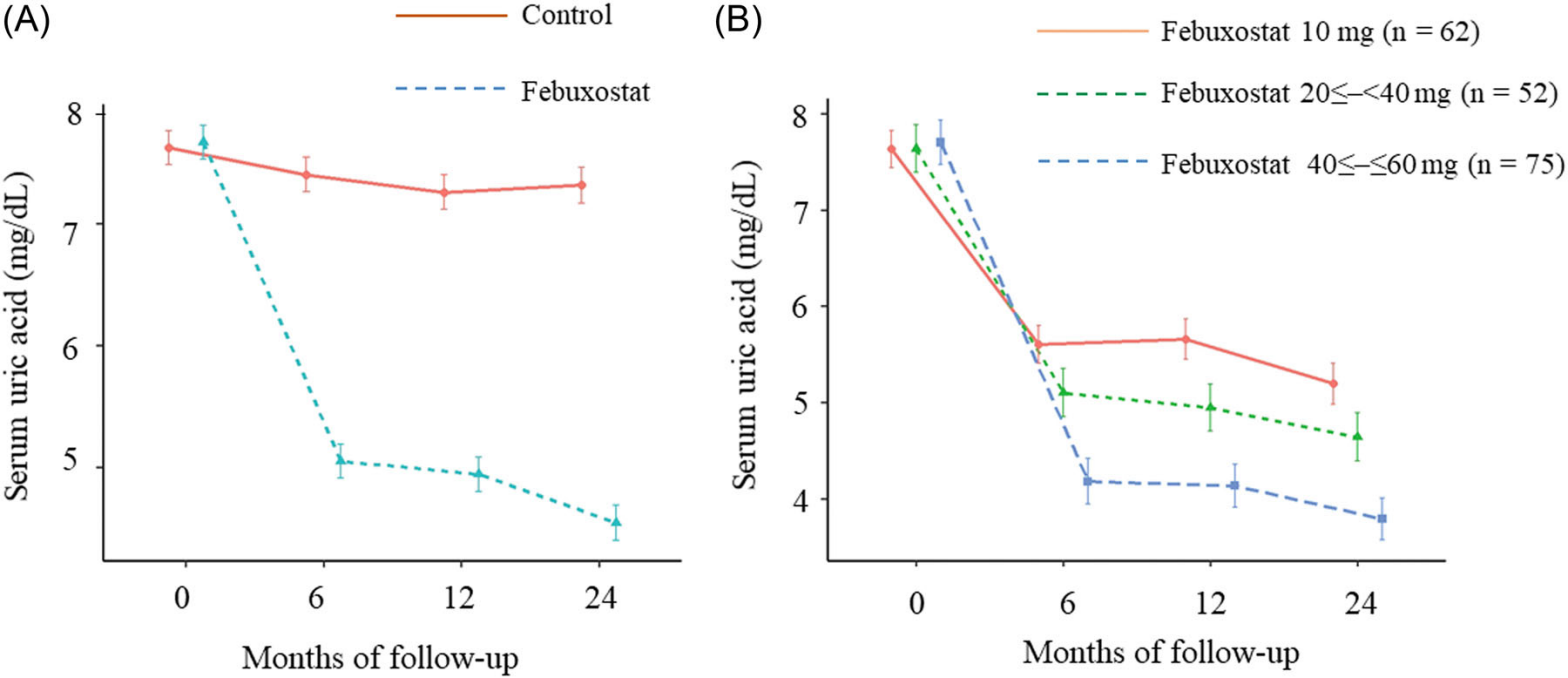
Changes in uric acid levels (A) between the febuxostat and control groups, overall, and (B) in the three subgroups based on the doses of febuxostat (10, ≤20 to <40 mg, ≤40 to ≤60 mg). The *p* value was ≤.001 at each time point (6, 12, and 24 months) in each figure.

The patients' characteristics are depicted in Table 1. There were no significant between‐group differences in the patient characteristics, such as the conventional coronary risk factors, comorbidity, and medications taken. Febuxostat significantly decreased the SUA levels from baseline to 24 months, as compared with those of the controls (*p* < .001 at 6, 12, and 24 months; Figure 2A). The mean doses of febuxostat at 6, 12, and 24 months were 22.8 ± 15.3, 23.5 ± 15.1, and 24.7 ± 15.9 mg, respectively. The SUA levels in the control group were mildly lowered (−0.3 mg/dL, *p* = .002 at 6 months, −0.4 mg/dL, *p* < .001 at 12 months, and −0.3 mg/dL, *p* < .001 at 24 months). The febuxostat‐associated reduction in SUA level increased with higher drug dosage (−2.6 mg/dL, *p* < .001 at 6 months, −2.7 mg/dL, *p* < .001 at 12 months, and −3.1 mg/dL, *p* < .001 at 24 months; Figure 2B).

**Table 1:** Patient characteristics.

	Control group	Febuxostat group	*p* Value	Missing (%)
No.	183	200		
Age (years)	70 (63, 76)	70 (63, 76)	.531	
Male/female	151/32	158/42	.384	
BMI	25.0 (22.6, 27.1)	24.6 (22.4, 26.9)	.216	5 (1.3)
Coronary risk factors (%)				
Current smoking	16 (9)	24 (12)	.419	0 (0)
Hypertension	166 (91)	175 (88)	.315	0 (0)
Dyslipidaemia	108 (59)	117 (59)	.918	0 (0)
Diabetes mellitus	69 (38)	79 (40)	.719	0 (0)
Comorbidity (%)				
Previous MI	15 (8)	22 (11)	.354	0 (0)
History of PCI	30 (16)	24 (12)	.217	0 (0)
History of CABG	12 (7)	11 (6)	.664	0 (0)
Heart failure	23 (13)	36 (18)	.141	0 (0)
Stoke	8 (4)	13 (7)	.361	0 (0)
Medications (%)				
Diuretics	52 (28)	58 (29)	.899	0 (0)
CCB	98 (54)	113 (57)	.562	0 (0)
ARB	109 (60)	111 (56)	.422	0 (0)
ACEI	19 (10)	21 (11)	.97	0 (0)
Beta receptor blocker	71 (39)	69 (35)	.383	0 (0)
Statins	89 (49)	95 (48)	.824	0 (0)
Ezetimibe	5 (3)	8 (4)	.494	0 (0)
Aspirin	66 (36)	64 (32)	.401	0 (0)

*Note*: Numbers are expressed as percentages, and values are expressed as medians with interquartile ranges.

Abbreviations: ACEI, angiotensin‐converting enzyme inhibitor; ARB, angiotensin II receptor blocker; BMI, body mass index; CABG, coronary artery bypass grafting; CCB, calcium‐channel blocker; MI, myocardial infarction; No., number; PCI, percutaneous coronary intervention.

### 3.2 Changes in MDA‐LDL level, LDL‐C level, and MDA‐LDL/LDL‐C ratio

**Figure 3:**
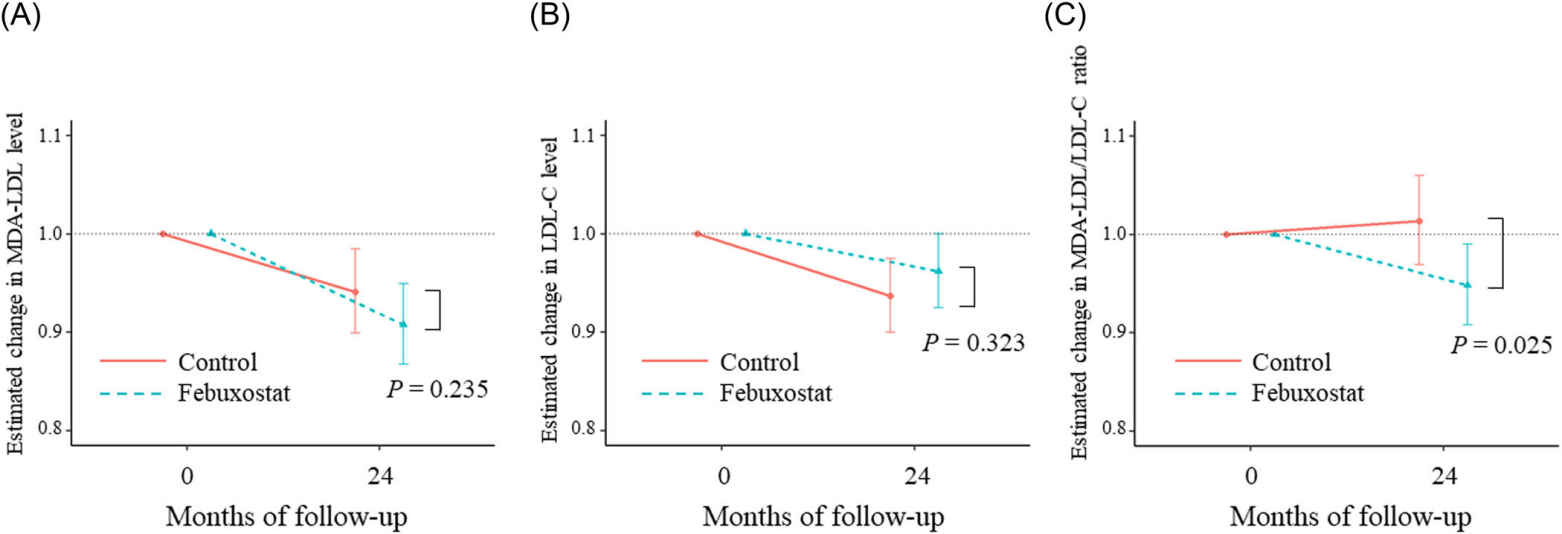
Association between time and estimated changes in the (A) MDA‐LDL level, (B) LDL‐C level, and (C) MDA‐LDL/LDL‐C ratio in the febuxostat and control groups. LDL‐C, low‐density lipoprotein cholesterol; MDA‐LDL, malondialdehyde‐modified low‐density lipoprotein.

MDA‐LDL levels, LDL‐C levels, and MDA‐LDL/LDL‐C ratios at baseline and 24 months in both treatment groups are depicted in Supporting Information: Table S1. These values were not different between the two groups, at the baseline and 24 months. Figure 3 displays the estimated changes in the MDA‐LDL level, LDL‐C level, and MDA‐LDL/LDL‐C ratio. There were no significant between‐group differences in the estimated changes in MDA‐LDL level (*p* = .235, Figure 3A) or LDL‐C (*p* = .323, Figure 3B). Nevertheless, there were significant between‐group differences in the estimated change in MDA‐LDL/LDL‐C ratio, (*p* = .025, Figure 3C). Overall (Supporting Information: Figure S1, left panels), the change in UA level (ΔUA) was positively related to the estimated changes in MDA‐LDL level (*p* = .020), but not to those in the LDL‐C level (*p* = .172) and MDA‐LDL/LDL‐C ratio (*p* = .147). No interaction was detected between the two groups (MDA‐LDL level: *p*_for interaction_ = 0.718, LDL‐C level: *p*_for interaction_ = 0.996, MDA‐LDL/LDL‐C ratio: *p*_for interaction_ = 0.795) (Supporting Information: Figure S1, right panels).

### 3.3 Relationship between the final dose of febuxostat and changes in MDA‐LDL level, LDL‐C level, and MDA‐LDL/LDL‐C

**Figure 4:**
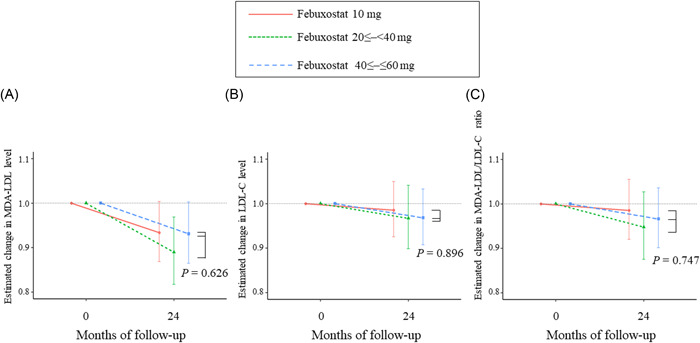
Association between time and the estimated changes in the (A) MDA‐LDL level, (B) LDL‐C level, and (C) MDA‐LDL/LDL‐C ratio according to the dose of febuxostat. LDL‐C, low‐density lipoprotein cholesterol; MDA‐LDL, malondialdehyde‐modified low‐density lipoprotein.

In the febuxostat group, 62, 52, and 75 patients had drug dosages of 10, ≤20 to <40 (20 mg, *n* = 47; 30 mg, *n* = 5), and ≤40 to ≤60 mg (40 mg, *n* = 64; 60 mg, *n* = 11), respectively. Changes in the MDA‐LDL level, LDL‐C level, and MDA‐LDL/LDL‐C ratio in this group, from the baseline value following the dose of febuxostat taken, are displayed in Figure 4. The dose of febuxostat did not significantly influence the changes in MDA‐LDL level (*p* = .626), LDL‐C level (*p* = .896), or MDA‐LDL/LDL‐C ratio (*p* = .747).

### 3.4 Relationship between the estimated change in mean CCA‐IMT and those in MDA‐LDL level, LDL‐C level, and MDA‐LDL/LDL‐C ratio

No significant relationship existed between the estimated change in mean CCA‐IMT and those in MDA‐LDL level (*p* = .927, Supporting Information: Figure S2A), LDL‐C level (*p* = .608, Supporting Information: Figure S2B), or MDA‐LDL/LDL‐C ratio (*p* = .805, Supporting Information: Figure S2C) in the overall population. In addition, there were no significant between‐group differences in the relationship between the estimated change in mean CCT‐IMT and those in MDA‐LDL level (*p*_for interaction_ = 0.972, Supporting Information: Figure S2A), LDL‐C (*p*_for interaction_ = 0.419, Supporting Information: Figure S2B), or MDA‐LDL/LDL‐C ratio, (*p*_for interaction_ = 0.727, Supporting Information: Figure S2C).

## 4 DISCUSSION

In the current subanalysis, we examined the effect of febuxostat, a selective XO inhibitor, on serum MDA‐LDL level, one of the oxidative stress markers, in patients having asymptomatic hyperuricemia and carotid arterial plaques. Generally, ΔUA was positively correlated with the changes in MDA‐LDL level, but not significantly correlated with the changes in LDL‐C level or MDA‐LDL/LDL‐C ratio. We also demonstrated that febuxostat reduced the MDA‐LDL/LDL‐C ratio compared with that of the controls, and it did not affect the MDA‐LDL or LDL‐C levels. Additionally, the dose of febuxostat did not affect this relationship, although the UA‐lowering effect of febuxostat is dose‐dependent. Patients included in the PRIZE study are frequently encountered in clinical practice, and we believe that long‐term febuxostat treatment for 2 years may decrease the MDA‐LDL/LDL‐C ratio in those patient populations.

MDA‐LDL has been recognized as one of the oxidative stress makers and has been implemented in clinical use.^6^, ^7^, ^8^ Nonetheless, it has been reported that this marker has been linked to LDL‐C level^26^ or the size of LDL particles.^27^ Therefore, MDA‐LDL/LDL‐C has also been employed as one of the oxidative stress makers^26^, ^27^, ^28^, ^29^, ^30^ and has been beneficial in observing the presence of significant atherosclerosis in patients with certain kinds of risk factors.^27^, ^31^, ^32^ In the current subanalysis, both the MDA‐LDL level and MDA‐LDL/LDL‐C ratio were implemented.

Our findings showed that ΔUA was positively related to the change in MDA‐LDL level, displaying the relationship between UA and oxidative stress,^33^ although the relationship between ΔUA and change in MDA‐LDL/LDL‐C ratio was insignificant. Contrarily, we demonstrated that febuxostat lowered the MDA‐LDL/LDL‐C ratio, but not the MDA‐LDL levels. It is uncertain about these discrepancies in the effect of febuxostat on MDA‐LDL levels and MDA‐LDL‐C ratio. As indicated above, there was no significant difference in changes in LDL‐C levels between the two groups, and it is unlikely that alterations because of LDL‐C itself were the cause. Additionally, overall, there was a significant relationship between the reduction in UA and change in MDA‐LDL level, but there were no group differences between the febuxostat and control groups, making it improbable that febuxostat lowered MDA‐LDL levels via UA reduction. The difference in the number of studied patients concerning MDA‐LDL level (*n* = 383) and MDA‐LDL/LDL‐C ratio (*n* = 358) in the current subanalysis may be one of the causes of these discrepancies. In the present subanalysis, it may be, therefore, difficult to say that febuxostat exerted an antioxidant effect because the impacts of febuxostat on MDA‐LDL level and MDA‐LDL/LDL‐C ratio were different. Nevertheless, the fact that febuxostat improved only the MDA‐LDL/LDL‐C ratio, which has been noted as a marker of atherosclerosis,^27^, ^31^, ^32^ may not rule out the possibility of an antioxidant effect, although weak. Further research is required to verify these outcomes concerning the importance of lowering only the MDA‐LDL/LDL‐C ratio and its impact on prognosis.

Although the antioxidant effect of febuxostat could not be fully elucidated from the present subanalysis, we considered potential reasons why compared with prior studies.^6^, ^12^, ^14^, ^16^, ^20^, ^21^ First, our research patients were asymptomatic hyperuricemic patients, although they had carotid artery plaques, and some had coronary artery disease and heart failure. The patients' pretreatment UA levels were approximately 7 mg/dL, which was not high. As the aforementioned reports^6^, ^12^, ^14^, ^16^, ^20^, ^21^ signify that the antioxidant effect of febuxostat may be stronger in patients with an increased oxidative stress state, it is likely that, in this research, the oxidative stress state of the patients might not be high. Second, it is also plausible that the control group in the current study was not treated medically, but with diet and exercise therapy, which might have enhanced their oxidative stress to some extent and further lowered MDA‐LDL levels^26^, ^28^, ^30^, ^34^ Third, our observation was 2 years, which was relatively long. In some of the above‐mentioned investigations, shorter observation periods were linked to enhanced oxidative stress indices.^6^, ^21^ It is possible that this trend may be because of the acute phase response in vivo, and that the MDA‐LDL level that was decreased during the acute phase may have been restored during the 2‐year observation period in this research, making the difference less apparent. In the present research, the MDA‐LDL levels were computed at baseline and 24 months; thus, the acute phase changes and detailed fluctuations in MDA‐LDL levels were not recorded in our investigation. Lastly, the dose of febuxostat was largely identified by the attending physician's judgment rather than by the protocol, and the final dose of febuxostat appeared to be lower than originally planned. These reasons may account for the less antioxidant effect of febuxostat shown in the current investigation.

We have noted the effect of febuxostat on the reduction in MDA‐LDL/LDL‐C ratio in the present research, and we believe that our results have the following strengths compared with prior reports^6^, ^12^, ^14^, ^16^, ^20^, ^21^: although the present investigation is a subanalysis, this is multicentre clinical research possessing a relatively large number of patients, MDA‐LDL was all measured in a centralized laboratory, and the impact was observed over a relatively long observation period of 2 years. In the current subanalysis, no significant correlation was observed between changes in MDA‐LDL or MDA‐LDL/LDL‐C and changes in CCA‐IMT over the 2‐year observation period, and no influence of these markers on the progression of carotid IMT was detected. Conversely, prior subanalyses of the PRIZE study demonstrated that febuxostat treatment improved arterial stiffness^35^ or left ventricular diastolic function,^36^ implying that the effect of febuxostat on reduction in MDA‐LDL/LDL‐C ratio detected in the current subanalysis may have contributed to such enhancements in cardiovascular parameters.

The current subanalysis has several limitations. First, the number of patients in this investigation was originally estimated for an assessment of changes in carotid IMT in the main study^22^; this number may be unsubstantial for the current subanalysis. Second, the study's protocol demonstrated that the maximum possible dose of febuxostat should be employed, but in reality, the dose was not elevated to the maximum level in many cases. Reportedly, the mean febuxostat daily dose was 22.8 mg at 24 months or termination for the full analysis set.^22^ This was largely the judgment of the attending physician, but we have pondered on the need to make frequent announcements to certify compliance with the protocol. Hence, the maximum effect of febuxostat may not have been accomplished in such cases. Third, the research protocol specified UA‐lowering drugs during the observation period, but there were no restrictions on drugs other than UA‐lowering drugs on UA and MDA‐LDL levels, which was left to the judgment of the attending physician, so there many cases may exist where drugs were added or discontinued. Drugs other than these UA‐lowering drugs may have affected the findings of this investigation. Fourth, although liquid chromatography is available for quantitative evaluation of febuxostat blood levels, it was not employed in this research. Finally, although there have been various oxidative stress markers that can be used clinically and several research have examined multiple indices,^14^ the present study used only one marker, that is, MDA‐LDL. Not all markers display similar changes; hence, it may not be possible to examine the oxidative stress status with a single marker. If possible, clinical investigations using numerous markers of oxidative stress should be performed in the future to confirm the antioxidant effect of febuxostat.

## 5 CONCLUSIONS

The current subanalysis of the PRIZE study may indicate a possibility that febuxostat, a selective XO inhibitor, reduces the MDA‐LDL/LDL‐C ratio, a potential marker of atherosclerosis and oxidative stress, in patients with asymptomatic hyperuricemia and carotid artery atherosclerosis. Nevertheless, this subanalysis is still subject to the challenges with the number of cases, the fact that the dose of febuxostat had not been increased, the heterogeneity of the involved patients, and the effects of drugs other than febuxostat and nonpharmacological treatments on UA and MDA‐LDL levels during the observation period. Further studies are required to validate our findings and elucidate the clinical antioxidant effect of febuxostat while considering those factors.

## AUTHOR CONTRIBUTIONS

**Hiroki Teragawa, Atsushi Tanaka, Toyoaki Murohara**, and **Koichi Node**: Conceptualization. **Hisako Yoshida**: Data curation. **Hisako Yoshida**: Formal analysis. **Koichi Node**: Funding acquisition. **Hiroki Teragawa, Atsushi Tanaka, Yuichi Fujii, Tomohiro Ueda, Shuichi Nomura, Toshiaki Kadokami, Hisashi Koide, Makoto Saito, Hiroaki Sano, Yasuko K. Bando, Toyoaki Murohara**, and **Koichi Node**: Investigation. **Hiroki Teragawa, Atsushi Tanaka, and Koichi Node**: Methodology. **Hiroki Teragawa, Atsushi Tanaka, Toyoaki Murohara**, and **Koichi Node**: Project administration. **Hisako Yoshida**: Resources. **Hisako Yoshida**: Software. **Atsushi Tanaka** and **Koichi Node**: Supervision. **Atsushi Tanaka** and **Koichi Node**: Validation. **Hisako Yoshida**: Visualization. **Hiroki Teragawa**: Writing—original draft. **Atsushi Tanaka, Yuichi Fujii, Hisako Yoshida, Tomohiro Ueda, Shuichi Nomura, Toshiaki Kadokami, Hisashi Koide, Makoto Saito, Hiroaki Sano, Yasuko K. Bando, Toyoaki Murohara**, and **Koichi Node**: Writing—review and editing. All authors have read and approved the final version of the manuscript.

## CONFLICTS OF INTEREST STATEMENT

Hiroki Teragawa received honoraria from Abbott Medical Japan, Bayer, Boehringer Ingelheim, Daiichi Sankyo, Kowa, Ono, Mitsubishi Tanabe, and Takeda. Atsushi Tanaka received honoraria from Boehringer Ingelheim and research funding from GlaxoSmithKline and Takeda. Toyoaki Murohara has received honoraria from AstraZeneca, Bayer, Boehringer Ingelheim, Japan, Daiichi Sankyo, Kowa, Novartis Pharma, Ono, and Sanwa Kagaku. Koichi Node has received honoraria from Astellas, AstraZeneca, Bayer Yakuhin, Boehringer Ingelheim Japan, Daiichi Sankyo, Eli Lilly Japan, Kowa, Mitsubishi Tanabe Pharma, Mochida Pharmaceutical, MSD, Novartis Pharma, Novo Nordisk Pharma, Ono Pharmaceutical, Otsuka, Teijin Pharma; Research grant from Asahi Kasei, Astellas, Mitsubishi Tanabe Pharma, Teijin Pharma, Boehringer Ingelheim Japan, Eli Lilly and Company, Novartis Pharma, Fuji Yakuhin, Mochida Pharmaceutical; Scholarship from Daiichi Sankyo Healthcare, Mitsubishi Tanabe Pharma, Teijin Pharma, Medtronic, Bayer Yakuhin. The remaining authors declare no conflict of interest.

## Supporting information

Supplementary information.

## Data Availability

The data that support the findings of this study are available upon reasonable request.
