# Supplementary material for: Effect of febuxostat on the level of malondialdehyde‐modified low‐density lipoprotein, an oxidative stress marker: A subanalysis of the PRIZE study
Source: Clin Cardiol. 2023 Mar 29;46(6):698–706. doi: 10.1002/clc.24014 (PMC10270262; doi:10.1002/clc.24014)
Supplement: Supplementary file 1 — Supplementary information. [file CLC-46-698-s001.pdf]

Table S1. MDA-LDL levels, LDL-C levels, and MDA-LDL/LDL-C ratios at baseline and 24M

	Control group	No.	Febuxostat group	No.
MDA-LDL				
Baseline	109 (85, 137)	183	108 (89, 133)	200
24M	106 (80, 132)	183	102 (77, 130)	200
LDL-C				
Baseline	109 97 (75, 120)	168	100 (79, 120)	190
24M	91 (71, 113)	168	95 (72, 119)	190
MDA-LDL/LDL-C ratio				
Baseline	1.14 (0.96, 1.48)	168	1.10 (0.90, 1.39)	190
24M	1.13 (0.92, 1.43)	168	1.07 (0.90, 1.33)	190

Values are expressed as medians and interquartile ranges. Abbreviations: LDL-C, low-density lipoprotein cholesterol; M, month; MDA-LDL, malondialdehyde-modified low-density lipoprotein; No., number.

Figure S1. Association between the change in UA and estimated changes in the (A) MDA-LDL level, (B) LDL-C level, and (C) MDA-LDL/LDL-C ratio.

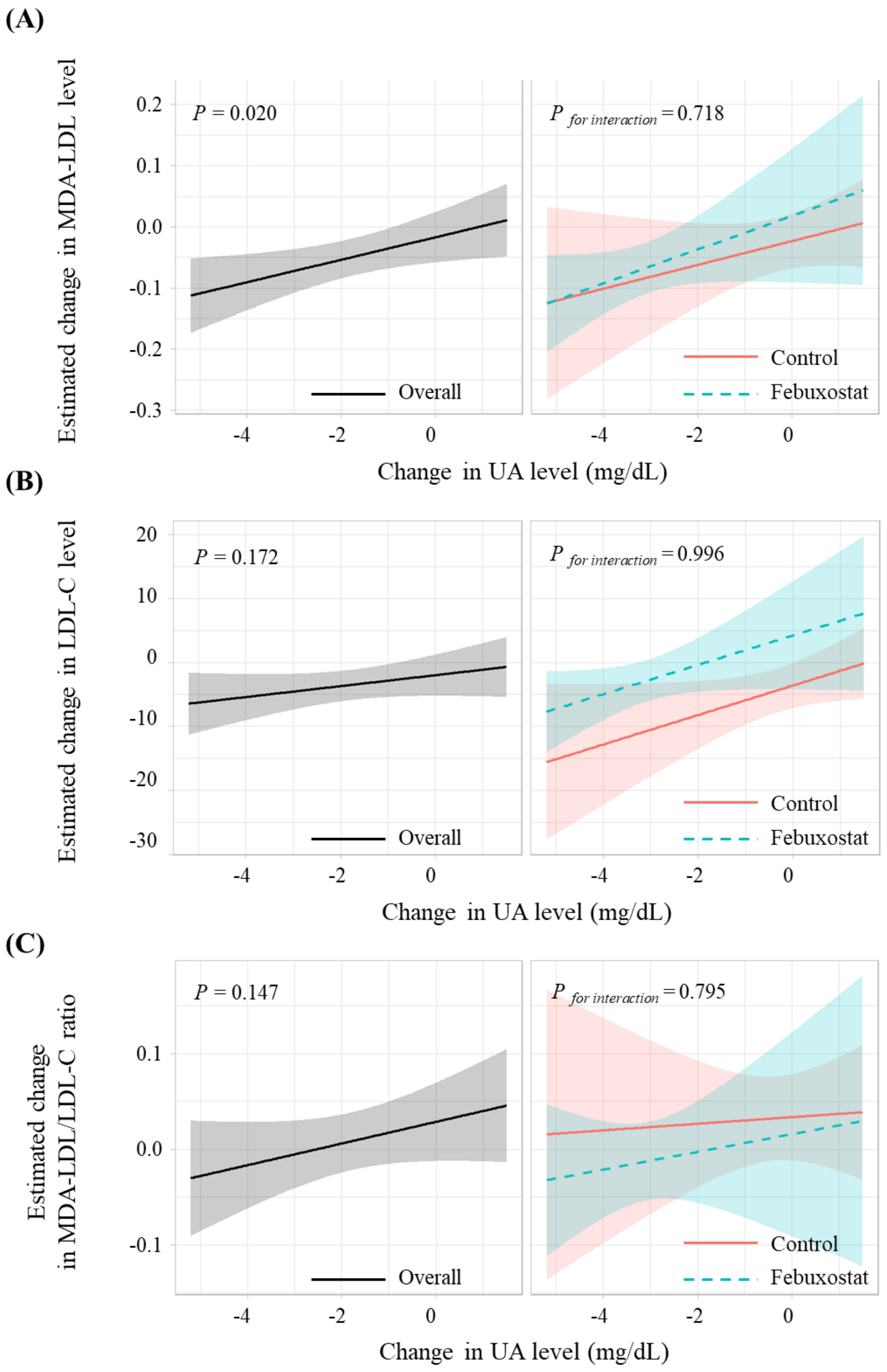

Overall population (left panels) and by treatment groups (right panels).

LDL-C, low-density lipoprotein cholesterol; MDA-LDL, malondialdehyde-modified low-density lipoprotein; UA, uric acid.

Figure S2. Association between the estimated change in mean CCA-IMT and the estimated changes in the (A) MDA-LDL level, (B) LDL-C level, and (C) MDA-LDL/LDL-C ratio.

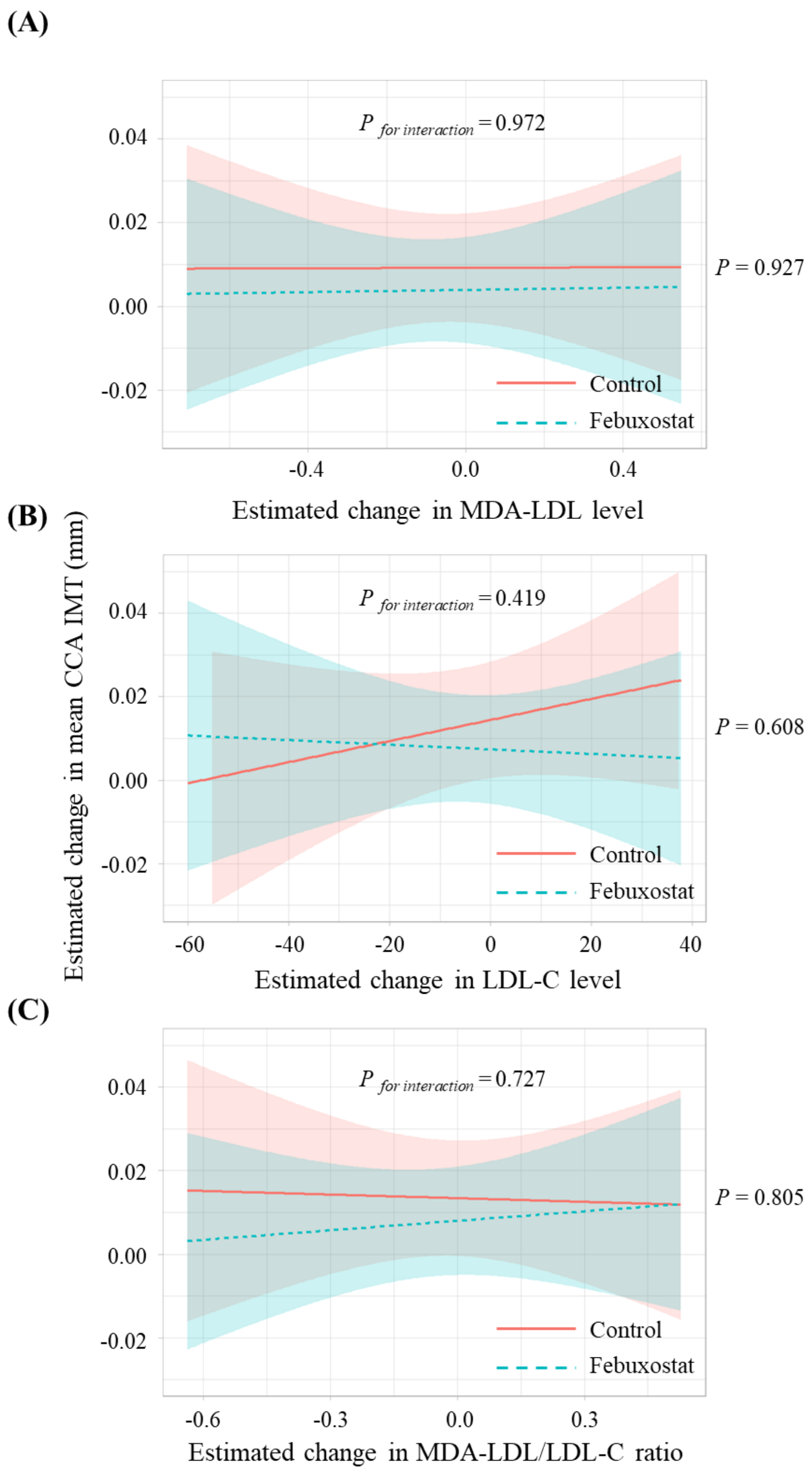

P values, shown to the right of each figure, indicate the relationship between the estimated changes in CCA-IMT and those in MDA-LDL level, LDL-C level, and MDA-LDL/LDL-C ratio in the overall population. $P_{for\ interaction}$, shown in the center of each figure, indicates a between-group difference in the relationships between the estimated change in CCA-IMT and those in MDA-LDL level, LDL-C level, and MDA-LDL/LDL-C ratio.

CCA, common carotid artery; IMT, intima-media thickness; LDL-C, low-density lipoprotein cholesterol; MDA-LDL, malondialdehyde-modified low-density lipoprotein.
